# Factors That Affect the Sensitivity of Imaging Modalities in Primary Hyperparathyroidism

**DOI:** 10.1155/2021/3108395

**Published:** 2021-02-20

**Authors:** Minting Zhu, Yang He, Tingting Liu, Bei Tao, Weiwei Zhan, Yifan Zhang, Jing Xie, Xi Chen, Hongyan Zhao, Lihao Sun, Jianmin Liu

**Affiliations:** ^1^Department of Endocrine and Metabolic Diseases, Ruijin Hospital, Shanghai Jiao Tong University School of Medicine, Shanghai Institute of Endocrine and Metabolic Diseases, and Shanghai Clinical Center for Endocrine and Metabolic Diseases, Shanghai 200025, China; ^2^Department of Ultrasonography, Ruijin Hospital, Shanghai Jiao Tong University School of Medicine, Shanghai 200025, China; ^3^Department of Nuclear Medicine, Ruijin Hospital, Shanghai Jiao Tong University School of Medicine, Shanghai 200025, China; ^4^Department of Pathology, Ruijin Hospital, Shanghai Jiao Tong University School of Medicine, Shanghai 200025, China; ^5^Department of Thyroid and Vascular Surgery, Ruijin Hospital, Shanghai Jiao Tong University School of Medicine, Shanghai 200025, China

## Abstract

**Background:**

Cervical ultrasound, ^99m^Tc-sestamibi single-photon emission computed tomography/computed tomography (^99m^Tc-MIBI SPECT/CT), and cervical CT are routinely used in preoperative localization of primary hyperparathyroidism (PHPT). However, false-negative imaging results are also frequently encountered in clinical practice. Exploring the factors that affect the sensitivity of these imaging modalities is important for the surgical management of PHPT patients.

**Methods:**

Clinical data of 352 PHPT patients hospitalized in our center from January 2011 to December 2015 were retrospectively collected to evaluate the sensitivity of 3 imaging modalities in the preoperative localization of parathyroid lesions. The ROC curve analysis was used to explore the clinical factors affecting the sensitivity of localization, and the cut-point(s) of related factors were determined.

**Results:**

^99m^Tc-MIBI SPECT/CT has the highest sensitivity among the localization modalities commonly used, reaching 91.1% (86.0%–94.8%). When the lengths of parathyroid lesions were ≤1.3 cm, the sensitivity of neck ultrasonography significantly decreased, while the sensitivity of ^99m^Tc-MIBI SPECT/CT decreased with parathyroid lesions ≤1.3 cm or serum PTH≤252 pg/ml. ^99m^Tc-MIBI SPECT/CT was less effective in localizing the hyperplasia lesions. Neck ultrasonography combined with ^99m^Tc-MIBI SPECT/CT can effectively improve the accuracy of preoperative localization of parathyroid lesions to 96.2% (92.7%–98.1%).

**Conclusions:**

Small parathyroid lesion and mild elevation of serum PTH would reduce the accuracy of parathyroid localization in PHPT patients.

## 1. Introduction

Primary hyperparathyroidism (PHPT) is an endocrine and metabolic disease caused by excessive secretion of parathyroid hormone (PTH) by parathyroid tissue, classically manifested as a group of clinical manifestations, including nausea, vomiting, kidney stones, and bone lesions [1]. Detection of asymptomatic PHPT patients is increasing in recent years, including China [2]. Normal parathyroid glands are located behind the thyroid gland in four quadrants. Single parathyroid adenoma, which accounts for 85%–90% of PHPT, is the most common pathological type of PHPT. Multiple glands involvement in PHPT is infrequent (5–10%) and can be manifested as multiple hyperplasia glands or multiple adenomas. Parathyroid carcinoma accounts for less than 1% in most European countries [3], but this rate is 3.9–6.0% in Asian patients [2, 4, 5].

Surgical treatment is the best treatment and the only way to cure PHPT. All symptomatic patients should consider surgery. The surgery indications of asymptomatic patients can refer to the guidelines from the Fourth International Workshop [6]. Although the location of the presumptive causative parathyroid gland is not a prerequisite to the diagnosis of PHPT and the criteria for surgical intervention, exact preoperative localization allows surgeons to plan a suitable surgical approach.

Imaging examinations are important in clinical practice [7]. Cervical ultrasound is the most commonly used and suitable imaging modality for parathyroid localization, with the advantages of lower cost and no radiation. However, it can poorly identify lesions behind the bone structure and esophagus, especially for ectopic lesions [8], and is highly operator dependent [9]. ^99m^Tc-sestamibi single-photon emission computed tomography/computed tomography (^99m^Tc-MIBI SPECT/CT), which scans both the neck and the mediastinum, is another widely used imaging modality with high sensitivity, especially in single adenoma [10]. Traditional radiology imaging technologies like computed tomography (CT) and magnetic resonance imaging (MRI) have limited utility. Four-dimensional (4D) CT has emerged, which is a multiphase, cross-sectional imaging study with the extra dimension referring to time [11]. The sensitivity of 4D CT is approximately 89.4% [12] and 73% for localizing the correct quadrant [13], which is much more sensitive than traditional neck CT. Meanwhile, a few new imaging technologies are involved in the positioning of parathyroid glands, including 4D-MRI, PET/CT, and PET/MR [14, 15].

Imaging modalities are not routinely used to confirm or exclude the diagnosis of PHPT but to find or locate hyperplasia or tumors. PHPT patients with negative imaging results but meet surgery indications should still be considered as candidates for surgery [16]. However, imaging plays an important role in operative planning, not to mention a negative or inconsistent preoperative localization imaging may reduce the patient's willingness to surgery. Therefore, it is necessary to explore the factors that lead to negative imaging results.

In the present study, we retrospectively evaluated the clinical and imaging data of PHPT patients in our center, in order to find out the factors affecting the positive rate of imaging modalities.

## 2. Materials and Methods

### 2.1. Patients

Clinical data of 352 PHPT patients hospitalized in our center (Shanghai, China) from January 2011 to December 2015 were retrospectively collected and analyzed. For those patients who were admitted several times, only the first admission was included. Diagnosis was made according to high serum PTH concentration and overt or mild hypercalcemia, with or without clinical manifestations of hypercalcemia [6]. Patients with secondary hyperparathyroidism, tertiary hyperparathyroidism, and multiple endocrine neoplasia were excluded.

To compare the difference of sensitivity among four imaging modalities (cervical ultrasonography, 99mTc-MIBI SPECT/CT, cervical contrast-enhanced CT scan, and 18F-FDG PET/CT). All patients were grouped according to the imaging exams applied. Patients who applied either the cervical ultrasonography or 99mTc-MIBI SPECT/CT were included in the fifth group named “USG or MIBI.” If either or both of the two exams successfully located the correct lesions, it was recorded as “true positive.”

This study was approved by the ethics committee of Ruijin Hospital affiliated to Shanghai Jiao Tong University School of Medicine (Ethics committee reference number: 2017–201). The patient's personal data have been secured.

### 2.2. Clinical Data and Biochemical Tests

The following clinical data were collected: (1) demographic data, including gender and age; (2) symptoms and signs; (3) preoperative biochemical indexes, including serum concentrations of creatinine, calcium, PTH, 25-hydroxy-vitamin *D* [25(OH)D], and urine concentrations of calcium in 24 hours, of which the highest serum calcium and PTH levels detected before treatment were recorded as the serum calcium and PTH before surgery; (4) preoperative localization imaging data, including cervical ultrasound, ^99m^Tc-MIBI SPECT/CT, and cervical contrast-enhanced CT; and (5) type of surgery, tumor size, and histological features.

Blood and urine biochemical tests were carried out to determine total serum calcium, phosphate, creatinine, total serum alkaline phosphatase (ALP), and 24-hour urinary calcium excretion. The albumin-corrected serum calcium level (mmol/L) was calculated according to the following formula: (40 − serum albumin concentration in grams per liter) × 0.02 + measured total serum calcium in millimoles per liter. The intact serum immunoreactive PTH level was measured using an intact immunoradiometric assay (normal range 15.0–68.3 pg/ml). The serum 25(OH)D concentration was measured with an enzyme immunoassay. The glomerular filtration rate was evaluated using the modification of diet in the renal disease formula. Bone mineral densities (BMDs) were measured using dual-energy X-ray absorptiometry (Lunar Prodigy; GE Medical Systems).

Whether the patients met the criterion for parathyroidectomy (PTX) was determined with reference to the indications for surgery based on the 2014 guidelines published after the fourth international workshop [6]: (1) all symptomatic PHPT and (2) asymptomatic patients meeting at least one of the following features: age<50 years; serum calcium level more than 0.25 mmol/L above the upper limits of normal; osteoporosis confirmed by BMD or fracture history; creatinine clearance<60 ml/min; and present stones or nephrocalcinosis.

The data of imaging examination was based on the records of the Hospital Information System (HIS): 336 patients completed cervical ultrasound, 276 completed ^99m^Tc-MIBI SPECT/CT, and 200 completed cervical contrast-enhanced CT. For neck ultrasound and cervical contrast-enhanced CT, positive results were determined if the imaging report mentioned the location of parathyroid lesions. For ^99m^Tc-MIBI SPECT/CT, any focus which showed tracer uptake along with retention on delayed imaging in the neck region or any potential site for the ectopic lesion on SPECT imaging, as well as a soft tissue mass in the corresponding position on CT imaging, was recorded as a positive result.

### 2.3. Imaging Modalities Used for Parathyroid Localization

#### 2.3.1. Cervical Ultrasound

It is completed by the Department of Ultrasound in our hospital, using the Italian Esaote MyLab 90, MyLab 60, and Philips Philips iU22 color Doppler ultrasound diagnostic instruments, line array high-frequency probe, and frequency 5∼13 MHz adjusting according to different conditions to optimize image quality. The patient took a supine position to scan the thyroid, parathyroid, and cervical lymph nodes.

#### 2.3.2. Cervical Contrast-Enhanced CT Scan

It is completed by the Department of Radiology in our hospital. The scanning instruments were lightspeed 16 and lightspeed VCT from GE of the United States. The patient was in a supine position with scanning from the oropharynx to the upper edge of the clavicle. The thickness of the scanning layer was 5 mm. The contrast agent was 100 ml of iopromide injection from Bayer of Germany. The injection rate was 2.5 ml/s and the scan was conducted 50–60 seconds after injection. The imaging diagnosis was independently performed by two experienced imaging physicians through PACS workstations.

#### 2.3.3. ^99m^Tc-MIBI SPECT/CT

It is completed by the Department of Nuclear Medicine, using dual-phase planar imaging and imaging equipment from the United States ADAC Corporation Vertex V60 single-photon emission computed tomography (SPECT) instrument. The patient took a supine position, after intravenous injection of ^99m^Tc-MIBI. A static acquisition (early imaging) was performed for 15 minutes and a delayed static acquisition (delayed imaging) was performed after 2 hours.

#### 2.3.4. Pathological Diagnosis

Pathological diagnosis was recorded according to the results of the paraffin pathology report in the Department of Pathology and divided into a parathyroid adenoma, parathyroid hyperplasia, parathyroid carcinoma, and other types. Patients with pathological diagnosis of “atypical adenoma” are classified into parathyroid adenoma. The pathological diagnosis of parathyroid cysts was based on a comprehensive analysis of pathological findings and clinical imaging findings. The diagnostic criteria for parathyroid carcinomas are based on the WHO criteria: (1) the tumor displays local invasion, such as perineural space invasion, capsular penetration with growth into adjacent tissue, vessel invasion, or invasion of vital organs or (2) the tumor has distant metastasis. As for the patients with parathyroid carcinoma undergoing primary surgery in other hospitals, pathological sections were sent to the Department of Pathology in our hospital for review and confirmed pathological diagnosis.

#### 2.3.5. Statistics

The data were analyzed using SPSS 19.0 statistical software package (SPSS). All *p* values were tested using a two-sided test, with *p* values ≤0.05 considered statistically significant. The variables were determined using the Kolmogorov–Smirnov test for normal distribution. All variables with normal distribution are expressed as “mean ± standard deviation,” and variables with nonnormal distribution are expressed as “median (lower quartiles-upper quartiles).” Ratios are expressed as “ratio (95% confidence interval).” When comparing between groups of variables, the normal distribution variable was analyzed by one-way analysis of variance or independent sample *t*-test. The nonnormal variable was tested by the rank-sum test (Mann–Whitney *U* test). The comparison between the sample rates was performed by chi-square test. ROC curve analysis was used to determine the effect of some clinical features on the sensitivity of imaging examinations. The cut-off point of relevant indicators was determined based on the index range when Youden's index was maximum.

## 3. Results

### 3.1. Clinical and Biochemical Data of Patients

From January 2011 to December 2015, a total of 352 patients were diagnosed with PHPT in the inpatient department of our center, including 263 female (74.72%) and 89 male patients (25.28%), with a female to male ratio of 2.96 : 1. The average age of all patients was 53.4 ± 12.5 years. A total of 247 (70.2%) out of the 352 PHPT patients had undergone surgical treatment. The clinical and laboratory data are summarized in Table 1.

Tumor length is recorded with histological length in the surgery group and the length measured by ultrasonography in nonsurgery group. Negative imaging studies failed to locate parathyroid lesion with imaging modalities including neck ultrasound, ^99m^Tc-MIBI SPECT/CT, and cervical CT.

### 3.2. Pathological Types in PHPT

Among 247 operated patients, 211 (85.43%) were pathologically diagnosed as parathyroid adenomas; 9 (3.64%) as parathyroid hyperplasia; 21 (8.50%) as malignant parathyroid carcinoma, and 6 (2.43%) as other pathological types, while two of them were adenomas with variable biological behaviors, one had adipose adenomas with atypical cells, other had simple parathyroid cysts, and parathyroid cysts had mixed lesions. A total of 12 patients with ectopic lesions accounted for 4.86% of the total number of patients. The most common ectopic site was the posterior sternum (4). Other ectopic sites included the thyroid gland (3), mediastinal (3), behind the clavicle (1), and upper the clavicle (1). Of the 21 patients with parathyroid carcinoma, 1 was an ectopic lesion located in the thyroid gland.

### 3.3. Sensitivity of 3 Imaging Modalities in Localizing Parathyroid Lesion

In the process of evaluating PHPT, the commonly used imaging examinations mainly include cervical ultrasound, ^99m^Tc-MIBI SPECT/CT, and cervical contrast-enhanced CT scans. In addition, 8 patients suspicious of malignancy with metastasis completed 18F-FDG PET/CT in our center as well. However, due to the limited cases and obvious selection bias, the sensitivity of PET/CT in localizing the lesion was not compared with other imaging modalities in this study. Positive is defined as a confirmed pathologic finding after surgery which suits a preoperative imaging study.

**Figure 1:**
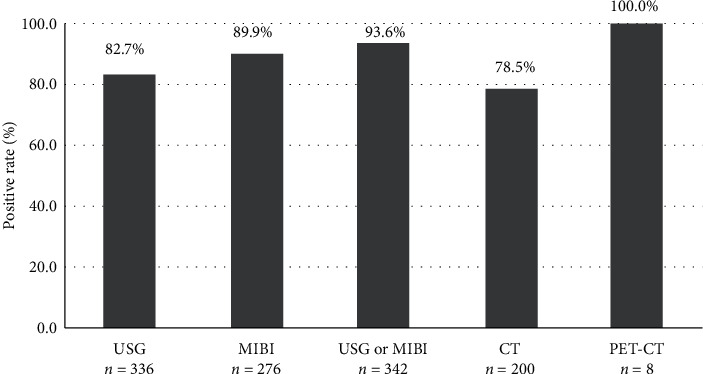
Positive rates in finding parathyroid lesions situated within the neck with different imaging methods. USG: cervical ultrasonography; MIBI: ^99m^Tc-MIBI SPECT/CT; CT: cervical contrast-enhanced CT scan; PET-CT: 18F-FDG PET/CT; USG or MIBI: applied either the cervical ultrasonography or ^99m^Tc-MIBI SPECT/CT.

Among a total of 336 patients who accomplished cervical ultrasound examination, the positive rate was 80.4% (75.8%–84.4%). Meanwhile, if excluding 10 patients with ectopic lesions situated outside the neck region, which were apparently unable to be localized by cervical ultrasound, the positive rate rose further to 82.7% (78.2%–86.5%). As for ^99m^Tc-MIBI SPECT/CT, the positive rate reached 89.9% (85.5%–93.0%) in finding parathyroid lesions situated within the neck region. Furthermore, all ectopic lesions were successfully localized by ^99m^Tc-MIBI SPECT/CT, which brought a 90.2% (86.2%–93.4%) positive rate for the whole group. The cervical CT scan was the least sensitive among those 3 imaging modalities. In 3 thoracic lesions detected by ^99m^Tc-MIBI SPECT/CT, thoracic CT only found 1. With the usage of neck ultrasound and ^99m^Tc-MIBI SPECT/CT in parallel, the positive rate came up to 93.6% (90.3%–95.8%) (Figure 1).

To calculate the sensitivity of the three localization imaging methods, pathological results were used as the gold standard. Therefore, the patients who were treated with surgery and could provide complete pathological results were included. Since a few ectopic lesions were located out of the normal scan range of some imaging modalities, patients with ectopic lesions located outside the neck were excluded. A total of 238 patients met the requirements. Lesion locations were noted as the left or right side of the neck, while ectopic lesions were recorded according to their original locations. “True positive” was recorded when the imaging examination matched the side of the location confirmed during surgery.

It was shown that the sensitivity of ^99m^Tc-MIBI SPECT/CT was the highest, reaching 91.1% (86.0%–94.8%) and that of the cervical ultrasound was 89.7% (85.0%–93.2%), while the sensitivity of cervical ultrasound along with ^99m^Tc-MIBI SPECT/CT could achieve 96.2% (92.7%–98.1%). Contrast-enhanced cervical CT got the lowest sensitivity (Table 2).

### 3.4. The Influence of Clinical Characteristics on Imaging Modalities

Through Youden's index in the ROC analysis, it was suggested that the lesion length (1.3 cm as cut-off point) and serum PTH (252 pg/ml as cut-off point) were helpful to distinguish between imaging-positive and imaging-negative patients in cervical ultrasound and ^99m^Tc-MIBI SPECT/CT, respectively. In order to further verify this result, we grouped patients who had undergone surgery according to the positive or negative imaging findings.

As shown in Table 3, the length of lesions in the ultrasound-positive group was significantly longer than that in the ultrasound-negative group (*p*=0.026). There was no significant difference in serum PTH levels between these two groups. No significant difference was found in lesion length and PTH between the MIBI-positive group and the negative group as well. There were no differences in serum calcium, ALP, Scr, 25-OHD concentrations, and MDRD between imaging positive and negative groups (data not shown).

After further grouping by lesion length and PTH values, it was revealed that when the lesion was ≤1.3 cm, the localization of the lesion(s) by cervical ultrasound and ^99m^Tc-MIBI SPECT/CT became difficult. The sensitivity of cervical ultrasound in lesions > 1.3 cm was 91.95% (86.61%–95.37%) but decreased to 76.92% (60.28%–88.29%) (*p*=0.018) when the lesion length was ≤1.3 cm. Meanwhile, the sensitivity of ^99m^Tc-MIBI SPECT/CT reduced from 93.94% (88.02%–97.15%) to 80.64% (61.94%–91.88%) (*p*=0.029) along with the decrease of lesion length (Table 4).

In addition, the sensitivity of ^99m^Tc-MIBI SPECT/CT was also affected by serum PTH concentration. When the serum PTH ≤ 252 pg/mL, the sensitivity of ^99m^Tc-MIBI SPECT/CT, as for 84.72% (73.88%–91.77%), was significantly lower than the high PTH group 95.00% (88.17%–98.14%) (*p*=0.022). The patient's age, gender, and BMI were not related to sensitivity, and no correlations were found in the serum creatinine, GFR-MDRD, serum calcium, and 25-OH vitamin D. The sensitivity of cervical CT was not affected by lesion length or serum PTH level (Table 5).

### 3.5. The Influence of Pathological Types on Imaging Modalities

As shown in Table 6, there was no significant difference in the sensitivity of neck ultrasound in the three pathological types of adenoma, hyperplasia, and carcinoma (85.7%–89.6%). The sensitivity of ^99m^Tc-MIBI SPECT/CT for adenoma was 96.9% (92.52%–98.85%), which was significantly higher than that for hyperplastic lesions. However, there was no significant difference comparing adenoma with carcinoma and carcinoma with hyperplasia as well. For adenomas and hyperplastic lesions, neck CT scans were not as sensitive as cervical ultrasound or ^99m^Tc-MIBI SPECT/CT and performed similarly between the three pathological types.

## 4. Discussion

The major finding of our study was that the sensitivity of ^99m^Tc-MIBI SPECT/CT was affected by the parathyroid lesion lengths and serum PTH levels, while that of cervical ultrasound was under the influence of lesion lengths, but not PTH levels.

In this study, ^99m^Tc-MIBI SPECT/CT is the most sensitive examination technique with a sensitivity of 91.1% (86.0%–94.8%), followed by 89.7% (85.0%–93.2%) for cervical ultrasound, and 82.1% (74.3%–88.0%) for cervical contrast-enhanced CT. In addition, ^99m^Tc-MIBI SPECT/CT successfully located all ectopic lesions (8 in total), which was much better than other modalities. In previous studies, the sensitivity of ^99m^Tc-MIBI scintigraphy with or without SPECT was 74%–97.7%, and the sensitivity of ultrasound was 64%–100% [17–21], which was consistent with the results of this study. It should be pointed out that our study uses “correct lateral” as a positive criterion, while some other studies use the quadrant of the lesion as a criterion. This may bring a higher sensitivity in our study than others. For example, Freudenberg et al. reported a ^99m^Tc-MIBI scintigraphy sensitivity of 74% [20], and Thanseer et al. reported a cervical ultrasound sensitivity of 69.3% [19] when applying quadrant-correct criterion. In addition, the use of cervical ultrasound together with ^99m^Tc-MIBI SPECT/CT can improve localization sensitivity to 96.2% (92.7%–98.1%), similar results in other studies can also be found [22]. Frank et al. pointed out that the use of two kinds of localization methods can achieve a better positioning diagnosis effect for PHPT patients with thyroid disease [23]. In addition, cervical ultrasound can help to determine concomitant thyroid disease, which is a vital component of evaluation before parathyroid surgery. Meanwhile, cervical CT performs not well enough in locating parathyroid lesions.

The most important finding in our study is that the length of the parathyroid lesion and serum PTH levels have a significant impact on the sensitivity of cervical ultrasound and ^99m^Tc-MIBI SPECT/CT. Furthermore, the cut points are identified. In our group of PHPT patients, there was a significant difference in the length of the lesions between the neck ultrasound positive and negative group, which indicates the larger the lesion, the better the accuracy of localization using cervical ultrasound. Our study further suggested that when lesion length is less than 1.3 cm, the sensitivity of cervical ultrasound is significantly reduced. Meanwhile, with ^99m^Tc-MIBI SPECT/CT applied, the lesions that were localized successfully seemed to be larger than those that failed, though the difference did not meet the statistical significance (*p*=0.09). Just as cervical ultrasound, a significant decrease in sensitivity of ^99m^Tc-MIBI SPECT/CT can be noticed when lesion length is less than 1.3 cm.

This is in keeping with the findings of some previous studies. Stack et al. found that parathyroid adenomas that weigh more than 350 mg are optimum for visualization by ^99m^Tc-MIBI SPECT/CT [24]. In addition, the sensitivity of ^99m^Tc-MIBI SPECT/CT could reach 92% when the weight of parathyroid adenoma is greater than 500 mg, and it decreased significantly to 64% in those lesions weighing less than 500 mg [25]. However, the weight of the lesion was not measured in our study; we only bring the length of the lesion as an indicator, which is easier to use in clinical practice.

On the other hand, the sensitivity of ^99m^Tc-MIBI SPECT/CT was also correlated with serum PTH level. In the case of serum PTH concentration less than 252 pg/ml, the false-negative rate of ^99m^Tc-MIBI SPECT/CT was significantly increased. A previous study showed that MIBI-negative parathyroid adenoma only present in patients with PTH values below 150 pg/ml [26], which indicated the influence of serum PTH on the sensitivity of ^99m^Tc-MIBI SPECT/CT. Parikshak et al. found that patients with PTH level over 160 pg/ml have greater than 90% sensitivity. They also suggested that patients with serum calcium over 2.8 mmol/L have higher sensitivity [27]. This correlation was not found in our study. The uptake and retention of MIBI in parathyroid tissue are relevant to oxyphil cells with rich mitochondria where intracellular MIBI sequestrate [28], which reflects the functional status of parathyroid glands [29]. Moreover, high PTH levels were reported to be related to positive uptake in early-phase ^99m^Tc-MIBI scintigraphy [30]. The results derived from our study also indicated that it was the elevation of serum PTH, but not calcium or other biochemical markers, which had an association with the sensitivity of ^99m^Tc-MIBI scintigraphy. The negative finding between serum calcium and the sensitivity of imaging modalities may in part derive from its larger fluctuation compared to PTH due to the hydration status in PHPT patients.

The median length of the parathyroid lesion in our country's PHPT patients ranged from 1.8 to 2.5 cm [2, 4]; therefore, most patients would have positioning confirmation with cervical ultrasound and ^99m^Tc-MIBI SPECT/CT. The serum PTH cut point (252 pg/ml) that affected the sensitivity of ^99m^Tc-MIBI SPECT/CT was basically the same as the median PTH in the present study (247.3 pg/mL). Therefore, attention should be paid to the risk of ^99m^Tc-MIBI SPECT/CT false negatives in those patients with relatively lower serum PTH. Given that the sensitivity of cervical ultrasound is not significantly associated with PTH, patients with relative lower PTH may consider using cervical ultrasound in priority. Cervical ultrasound along with MIBI should be recommended in patients with small parathyroid lesions, even though they have lower sensitivity. If they fail to localize, referral to more experienced sonographers or clinical centers is an option, since ultrasound is highly dependent on operator experience. Novel techniques including but not limited to 4D-CT, ^11^C-choline PET/CT, and ^18^F-flurocholine PET/CT have been reported to have satisfactory high sensitivities, which are worth trying. In the end, localization should not be used to decide if a patient should proceed with surgery [31]. Bilateral exploratory surgery should always be taken into consideration in patients without confirmed localization.

Although ^99m^Tc-MIBI SPECT/CT has high sensitivity and performs well in locating ectopic parathyroid, its sensitivity for parathyroid hyperplasia is only 60%, much lower than that of cervical ultrasound (87.5%). It also suggests that ^99m^Tc-MIBI SPECT/CT is less sensitive to parathyroid hyperplasia than parathyroid adenoma, which is in good accordance with previous studies [22, 25]. Torregrosa et al. have found that, in secondary hyperparathyroidism, the uptake of ^99m^Tc-MIBI is correlated with the cell cycle phases. Low-grade uptake has a relation with the G(0) phase and higher uptake with G(2)+S phase [32], suggesting that the unsynchronized cell cycles between multiple lesions may take part as well in rising localization difficulty for ^99m^Tc-MIBI scintigraphy in PHPT. Other causes of false negatives in parathyroid ^99m^Tc-MIBI scintigraphy may also be due to ectopic parathyroid lesions outside the scope of routine examination; along with thyroiditis or hyperthyroidism, which accelerate the metabolism of ^99m^Tc-MIBI [23]. In addition, when the lesion shows the appearance of a large cystic one, the uptake of ^99m^Tc-MIBI is reduced, and the lesion appears less or equal radioactivity to the thyroid [33]. In the end, it needs to be emphasized that localization should not be used to decide if a patient should proceed with surgery. Bilateral exploratory surgery should always be taken into consideration in patients without clear preoperation localization.

On the other hand, through pathological grouping, there is no significant difference in the sensitivity of cervical ultrasound, ^99m^Tc-MIBI SPECT/CT, and cervical contrast-enhanced CT in parathyroid adenoma and carcinoma. It suggests that the commonly used imaging methods do not perform well enough in distinguishing benign parathyroid tumors from maligant ones.

This study has several limitations. In this study, there were no PHPT patients with low to borderline elevated PTH, which may lead to a better sensitivity of imaging modalities than in other regions which have more normohormonal PHPT. A standard one-phase contrast-enhanced CT was used in this study, but not the currently preferred method utilizing pre- and postcontrast techniques with the assessment of contrast washout. Lack of 4D CT/MRI data may underestimate the function of radiographic methods in localizing parathyroid. The data on the volume of tumors and the BMD measured at the distal radium were not available.

## 5. Conclusions

In PHPT, ^99m^Tc-MIBI SPECT/CT has the best sensitivity in localizing parathyroid lesions. Shorter parathyroid lesion length and mild elevation of serum PTH are the potential reasons for decreased sensitivity of localization examinations.

## Figures and Tables

**Table 1:** Clinical features and laboratory test results in PHPT patients.

	Total patients
Number	352
Age, y	53.4 ± 12.5
Sex (female : male)	2.96 : 1
Duration of disease, y	1 (0.2–4.0)
Nephrolithiasis (*n*, %)	136 (38.64%)
Fracture (*n*, %)	30 (8.52%)
Serum calcium, mmol/L	2.76 ± 0.31
Serum phosphate, mmol/L	0.88 ± 0.20
Serum albumin, g/L	38.25 ± 3.72
Albumin-corrected serum calcium, mmol/L	2.79 ± 0.32
Serum PTH, pg/mL	247.3 (150.0–537.9)
Serum ALP, IU/L	93 (68–142)
Serum 25(OH)D, nmol/L	27.24 (18.05–38.68)
24 h urinary calcium, mmol/24 h	6.03 (4.35–8.09)
Serum creatinine, *μ*mol/L	63 (54–79)
MDRD, ml/min/1.73 m^2^	99 (80–113)
BMD L1-L4, g/cm^2^	0.93 ± 0.20
T-score L1-L4	−1.58 ± 1.65
BMD femoral neck, g/cm^2^	0.76 ± 0.14
T-score femoral neck	−1.30 ± 1.16
Osteoporosis (T ≦ −2.5) (%)	36.27%
Negative imaging results (*n*, %)	18 (5.11%)
Tumor length (cm)	1.8 (1.3–2.7)

PTH: parathyroid hormone; ALP: alkaline phosphatase; 25(OH)D: 25-hydroxyvitamin *D*; MDRD: modification of diet in renal disease; BMD: bone mineral density.

**Table 2:** Sensitivity of different imaging methods in localizing parathyroid lesion in PHPT.

	Pathology (no.)	Imaging (no.)	Sensitivity (95% CI)
USG	234	210	89.7% (85.0%–93.2%)
MIBI	180	164	91.1% (86.0%–94.8%)
USG or MIBI	238	229	96.2% (92.7%–98.1%)
CT	134	110	82.1% (74.3%–88.0%)
PET-CT	7	7	100.0% (56.1%–100.0%)

USG: cervical ultrasonography; MIBI: ^99m^Tc-MIBI SPECT/CT; CT: cervical contrast-enhanced CT scan; PET-CT: 18F-FDG PET/CT; USG or MIBI: applied either the cervical ultrasonography or ^99m^Tc-MIBI SPECT/CT.

**Table 3:** Influence of clinical features on cervical ultrasonography and 99mTc-MIBI SPECT/CT in searching parathyroid lesions.

	USG positive *n* = 210	USG negative *n* = 24	MIBI positive *n* = 164	MIBI negative *n* = 16	*P* value (USG)	*P* value (MIBI)
Sex, male (%)	29.05%	28.00%	28.74%	43.75%	0.913	0.211
Age (y)	52.1 ± 12.4	56.3 ± 10.8	51.9 ± 12.6	55.8 ± 8.9	0.103	0.228
BMI (kg/m^2^)	22.7 ± 3.5	23.5 ± 3.5	22.7 ± 3.6	23.1 ± 4.4	0.260	0.699
Lesion length^†^ (cm)	2.0 (1.5–3.0)	1.8 (1.0–2.5)	2.0 (1.5–3.0)	1.8 (1.0–2.3)	0.026^*∗*^	0.090
≤1.3 cm	15.79%	39.13%	16.78%	42.86%	0.018^*∗*^	0.029^*∗*^
(%, *n*)	(30/190)	(9/23)	(25/149)	(6/14)		
>1.3 cm	84.21%	60.87%	83.22%	57.14%		
(%, *n*)	(160/190)	(14/23)	(124/149)	(8/14)		
PTH^†^ (pg/mL)	288.2 (167.4–667.7)	264.6 (163.8–673.6)	328.4 (177.2–738.1)	210.2 (135.2–664.6)	0.873	0.147
≤252 pg/mL	43.09%	45.00%	39.10%	68.75%	0.869	0.022^*∗*^
(%, *n*)	(81/188)	(9/20)	(61/156)	(11/16)		
>252 pg/mL	56.91%	55.00%	60.90%	31.25%		
(%, *n*)	(107/188)	(11/20)	(95/156)	(5/16)		

^*∗*^ *p* < 0.05. ^†^Lesion length and PTH were shown with median (higher quartile-lower quartile).

**Table 4:** Sensitivity of imaging methods in two groups classified by lesion length.

Lesion length (cm)	USG (sensitivity, *n*)	MIBI (sensitivity, *n*)	CT (sensitivity, *n*)
>1.3	91.95% (160/174)	93.94% (124/132)	83.0% (83/100)
≤1.3	76.92% (30/39)^*∗*^	80.64% (25/31)^*∗*^	73.68% (14/19)

^*∗*^ *p* < 0.05 between lesion length groups.

**Table 5:** Sensitivity of imaging methods in two groups classified by serum PTH.

PTH (pg/ml)	USG (sensitivity, *n*)	MIBI (sensitivity, *n*)	CT (sensitivity, *n*)
>252	90.68% (107/118)	95.00% (95/100)	85.00% (68/80)
≤252	89.01% (81/91)	84.72% (61/72)^*∗*^	76.00% (38/50)

^*∗*^ *p* < 0.05 between PTH groups. USG: cervical ultrasonography; MIBI: ^99m^Tc-MIBI SPECT/CT; CT: cervical contrast-enhanced CT scan.

**Table 6:** Sensitivity of imaging methods in different PHPT pathological types.

	Adenoma	Hyperplasia	Carcinoma
Number	211	9	21
USG	89.6% (181/202)	87.5% (7/8)	85.7% (18/21)
MIBI	96.9% (156/161)^*∗*^	60.0% (3/5)	93.3% (14/15)
CT	80.9% (93/115)	50.0% (2/4)	92.9% (13/14)
PET/CT	100.0% (4/4)		100.0% (3/3)

USG: cervical ultrasonography; MIBI: ^99m^Tc-MIBI scintigraphy; CT: cervical contrast-enhanced CT scan; PET-CT: 18F-FDG PET/CT. ^*∗*^*p* < 0.05 adenoma versus hyperplasia.

## Data Availability

The data used to support the findings of this study are available from the corresponding author upon request.

## References

[1] Pallan S., Rahman M. O., Khan A. A. (2012). Diagnosis and management of primary hyperparathyroidism. *BMJ*.

[2] Zhao L., Liu J.-m., He X.-Y. (2013). The changing clinical patterns of primary hyperparathyroidism in Chinese patients: data from 2000 to 2010 in a single clinical center. *The Journal of Clinical Endocrinology & Metabolism*.

[3] Marcocci C., Cetani F. (2011). Primary hyperparathyroidism. *New England Journal of Medicine*.

[4] Sun B., Guo B., Wu B. (2018). Characteristics, management, and outcome of primary hyperparathyroidism at a single clinical center from 2005 to 2016. *Osteoporosis International*.

[5] Kobayashi T., Sugimoto T., Chihara K. (1997). Clinical and biochemical presentation of primary hyperparathyroidism in kansai district of Japan. *Endocrine Journal*.

[6] Bilezikian J. P., Brandi M. L., Eastell R. (2014). Guidelines for the management of asymptomatic primary hyperparathyroidism: summary statement from the Fourth International Workshop. *The Journal of Clinical Endocrinology & Metabolism*.

[7] Haddouche A., Bellanne‐Chantelot C., Rod A. (2020). Liver adenomatosis in patients with hepatocyte nuclear factor‐1 alpha maturity onset diabetes of the young (HNF1A ‐MODY): clinical, radiological and pathological characteristics in a French series. *Journal of Diabetes*.

[8] Kitaoka M. (2003). Ultrasonographic diagnosis of parathyroid glands and percutaneous ethanol injection therapy. *Nephrol Dial Transplant*.

[9] Boccalatte L. A. (2019). Usefulness of 18F-fluorocholine positron emission tomography-computed tomography in locating lesions in hyperparathyroidism: a systematic review. *JAMA Otolaryngology–Head & Neck Surgery*.

[10] Madorin C. A., Owen R., Coakley B. (2013). Comparison of radiation exposure and cost between dynamic computed tomography and sestamibi scintigraphy for preoperative localization of parathyroid lesions. *JAMA Surgery*.

[11] Hoang J. K., Reiman R. E., Nguyen G. B. (2015). Lifetime attributable risk of cancer from radiation exposure during parathyroid imaging: comparison of 4D CT and parathyroid scintigraphy. *American Journal of Roentgenology*.

[12] Cheung K., Wang T. S., Farrokhyar F., Roman S. A., Sosa J. A. (2012). A meta-analysis of preoperative localization techniques for patients with primary hyperparathyroidism. *Annals of Surgical Oncology*.

[13] Kluijfhout W. P., Pasternak J. D., Beninato T. (2017). Diagnostic performance of computed tomography for parathyroid adenoma localization; a systematic review and meta-analysis. *European Journal of Radiology*.

[14] Quak E., Blanchard D., Houdu B. (2018). F18-choline PET/CT guided surgery in primary hyperparathyroidism when ultrasound and MIBI SPECT/CT are negative or inconclusive: the APACH1 study. *European Journal of Nuclear Medicine and Molecular Imaging*.

[15] Purz S., Kluge R., Barthel H. (2013). Visualization of ectopic parathyroid adenomas. *New England Journal of Medicine*.

[16] Wilhelm S. M., Wang T. S., Ruan D. T. (2016). The American association of endocrine surgeons guidelines for definitive management of primary hyperparathyroidism. *JAMA Surgery*.

[17] Tublin M. E., Pryma D. A., Yim J. H. (2009). Localization of parathyroid adenomas by sonography and technetium Tc 99m sestamibi single-photon emission computed tomography before minimally invasive parathyroidectomy. *Journal of Ultrasound in Medicine*.

[18] Nafisi Moghadam R. (2017). Comparative diagnostic performance of ultrasonography and 99mTc-sestamibi scintigraphy for parathyroid adenoma in primary hyperparathyroidism; systematic review and meta- analysis. *Asian Pacific Journal of Cancer Prevention*.

[19] Thanseer N., Bhadada S. K., Sood A. (2017). Comparative effectiveness of ultrasonography, 99mTc-sestamibi, and 18F-fluorocholine PET/CT in detecting parathyroid adenomas in patients with primary hyperparathyroidism. *Clinical Nuclear Medicine*.

[20] Freudenberg L. S., Frilling A., Sheu S.-Y., Görges R. (2006). Optimizing preoperative imaging in primary hyperparathyroidism. *Langenbeck’s Archives of Surgery*.

[21] Berczi C., Mezõsi E., Galuska L. (2002). Technetium-99m-sestamibi/pertechnetate subtraction scintigraphy vs ultrasonography for preoperative localization in primary hyperparathyroidism. *European Radiology*.

[22] Xue J., Liu Y., Ji T. (2018). Comparison between technetium-99m methoxyisobutylisonitrile scintigraphy and ultrasound in the diagnosis of parathyroid adenoma and parathyroid hyperplasia. *Nuclear Medicine Communications*.

[23] Frank E. (2018). *Preoperative imaging for parathyroid localization in patients with concurrent thyroid disease: a systematic review*.

[24] Stack B. C., Moore E. R., Belcher R. H., Spencer H. J., Bodenner D. L. (2012). Hormone, relationships of parathyroid gamma counts, and adenoma mass in minimally invasive parathyroidectomy. *Otolaryngology-Head and Neck Surgery*.

[25] Andersen T. B., Aleksyniene R., Boldsen S. K., Gade M., Bertelsen H., Petersen L. J. (2018). Contrast-enhanced computed tomography does not improve the diagnostic value of parathyroid dual-phase MIBI SPECT/CT. *Nuclear Medicine Communications*.

[26] Cordes M., Dworak O., Papadopoulos T., Coerper S., Kuwert T. (2018). MIBI scintigraphy of parathyroid adenomas: correlation with biochemical and histological markers. *Endocrine Research*.

[27] Parikshak M., Castillo E. D, Conrad M. F, Talpos G. B (2003). Impact of hypercalcemia and parathyroid hormone level on the sensitivity of preoperative sestamibi scanning for primary hyperparathyroidism. *The American Surgeon*.

[28] Melloul M., Paz A., Koren R., Cytron S., Feinmesser R., Gal R. (2001). 99mTc-MIBI scintigraphy of parathyroid adenomas and its relation to tumour size and oxyphil cell abundance. *European Journal of Nuclear Medicine*.

[29] Pons F., Torregrosa J. V., Fuster D. (2003). Biological factors influencing parathyroid localization. *Nuclear Medicine Communications*.

[30] Kushchayeva Y. S., Tella S. H., Kushchayev S. V., Van Nostrand D., Kulkarni K. (2019). Comparison of hyperparathyroidism types and utility of dual radiopharmaceutical acquisition with Tc99m sestamibi and 123I for localization of rapid washout parathyroid adenomas. *Osteoporosis International*.

[31] Goldfarb M., Singer F. R. (2020). *Recent advances in the understanding and management of primary hyperparathyroidism, F1000Res*.

[32] Torregrosa J. V. (2000). (99m)Tc-sestamibi scintigraphy and cell cycle in parathyroid glands of secondary hyperparathyroidism. *World Journal of Surgery*.

[33] Pontikides N., Karras S., Kaprara A. (2012). Diagnostic and therapeutic review of cystic parathyroid lesions. *Hormones*.

